# The Undergraduate Genomics Research Initiative

**DOI:** 10.1371/journal.pbio.0050141

**Published:** 2007-05-15

**Authors:** Cheryl A Kerfeld, Robert W Simons

## Abstract

The inherent blend of wet laboratory experience and in silico experimentation of genomics research makes it an ideal model to illustrate the interdisciplinary nature of life sciences research today.

Modern scientific advances have transformed life sciences research but have had little influence on undergraduate training, leaving an unprecedented gap between teaching and research. Consequently, a consensus is building around the need to reform undergraduate life sciences education [1–3]. Students need to embrace scientific discovery directly, make connections across an otherwise diverse curriculum, learn to manage and interpret today's vast amounts of data, practice using computers to control instrumentation and analyze experiments, and greatly improve quantitative reasoning. Moreover, they need to appreciate that modern life sciences research is increasingly carried out by interdisciplinary teams of scientists—yet teamwork is alien to the highly competitive undergraduate life sciences culture. Indeed, a recent poll by the Science Advisory Board, an international “electronic community” of scientists and physicians, determined that “poor interpersonal skills are hampering the careers of young researchers;” they have difficulty working in teams [4].

Research experience is widely recognized as an ideal way to achieve many of these reforms simultaneously while giving students the chance to experience the emotions, challenges, and satisfactions inherent in doing research. Providing research experience to all life sciences students, however, presents a seemingly intractable scaling problem. Carefully guided by a faculty member, graduate student, or post-doc, the typical undergraduate research project is labor intensive—requiring hypotheses-driven experiments, intense data evaluation, and, often, many different techniques. Although excellent training occurs, most faculty members can productively mentor only one or few undergraduates in this kind of research. Even a major research university such as the University of California Los Angeles (UCLA) can accommodate only about 20% of qualified students in this way.

The inherent blend of wet laboratory experience and in silico experimentation of genomics research makes it an ideal model to illustrate the interdisciplinary nature of life sciences research today.

Clearly, a new approach to undergraduate research is required, one that reflects modern life sciences research yet enables large numbers of students to participate. We sought to address this problem by developing a microbial genome sequencing project specifically for undergraduates, The Undergraduate Genomics Research Initiative, UGRI [5]. The UGRI addresses the problems of scope and scale in two ways. First, to serve as a hub for this collaborative research network, we created a new interdisciplinary research course, LS187, “Principles and Practices of Genomics Research,” that blends topics in molecular biology, microbiology, evolution, bacterial physiology, genomics, physics, and bioinformatics in the sequencing and analysis of a microbial genome. Second, we dissolved the traditional boundary between coursework and research by enabling large numbers of students in traditional courses to directly contribute individual research effort to the LS187 hub course, in turn receiving raw data for their own analysis and report. Thousands of students annually participate in this collaborative research project. Here we describe the structure and outcomes of the first three years of the UGRI, focusing on the course, LS187. We also discuss how other institutions can adapt this collaborative research approach.

## Bringing Research into the Classroom

**Figure 1:**
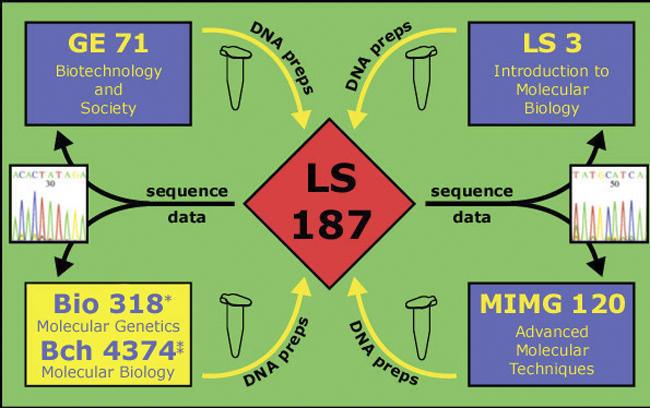
LS187 Is the Hub of a Group of Courses Dedicated to the Sequencing of a Microbial Genome Associated courses at UCLA (Table S1) are shown in blue; Biology 318 is taught at St John's University/College of St. Benedict in Collegeville MN. Bch 4374 is taught at the University of Missouri, Columbia, and Bio3027 is taught at the University of Minnesota, Crookston.

The core course of the UGRI is LS187 (Figure 1, Figure S1, Protocol S1)—a research course (Protocol S1) that is dedicated to the sequencing and analysis of the genome of Ammonifex degensii [6], a thermophilic chemolithoautotroph that uses a variety of energy generating pathways. We chose this lesser-known microbe to minimize the risk that the work would be “scooped,” but A. degensii nevertheless has interesting phenotypic traits suited to illustrating concepts across the life sciences curriculum.

Unlike the traditional role of students as knowledge consumers, UGRI undergraduates are knowledge producers. An overwhelming majority of students report that knowing their sequence data is available to other researchers on the NCBI website makes their work more interesting and motivates them to do their best.

After completing the course for the first time (LS187A), many students reenroll in LS187B and subsequently LS187C. These students learn more advanced skills relating to sequence analysis and annotation, and importantly, they act as mentors, providing one-on-one guidance in wet lab techniques for the LS187A students.

The LS187 course is modeled after a research group: each week, students have a lab meeting to discuss the latest results and troubleshoot any problems. LS187BC students take leadership roles in these discussions, using their experience to trace the symptom of a problem to its probable cause, and to report discoveries. Because the LS187 laboratory is open for just four hours daily, students must take a collaborative, organized approach to best make use of this time: some students prepare the sequencing gels, some set up the PCR reactions, and others analyze the previous day's results or work on expanding the genomic library (Protocol S1).

The genomic sequence data generated by the LS187 students are returned as FASTA and Trace files to the students in associated courses for a quality check and bioinformatic analysis. Students in LS187 also analyze the data and record the results of homology searches in the project database. Additionally, they assist in preparing the data for deposition to the National Center for Biotechnology Information (NCBI) Trace File Archive (http://www.ncbi.nlm.nih.gov/Traces/trace.cgi).

LS187A students take weekly seminars on topics related to genomics research (Table 1), which provide the ideal raw material for making connections across the undergraduate curriculum. The seminars offer students a chance to revisit topics learned in other life sciences courses (e.g., redox chemistry, PCR, autotrophy). And by working through how *E*-values (the measure of the significance of the alignment between sequences) are calculated by the BLAST algorithm [7], for example, students learn how biological processes (such as DNA sequence insertion and deletion events) can be modeled mathematically. Other seminars focus on the physics underlying the DNA sequencer and arrangement of its optical components to show students how the instrumentation works. Weekly quizzes on these topics help ensure that the students have a thorough understanding of both the methodological and theoretical basis of their research.

**Table 1:**
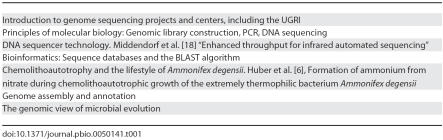
Seminar Topics in LS187A

## The Student Experience

The inherent blend of wet laboratory experience and in silico experimentation of genomics research makes it an ideal model to illustrate the interdisciplinary nature of life sciences research today. LS187 teaches students how to use computers and algorithms to acquire and interpret data. For example, students use a base-calling program to read DNA sequences from the virtual gels and, in doing so, discover that even robust programs are not infallible, especially in cases where the sequencing reaction is poor or the gel is imperfect. Grappling with challenging data helps students acquire the skills to manipulate program parameters to fit the needs of the experiment instead of blindly relying on default settings [8,9].

Furthermore, students frequently accept information from the internet uncritically; in research, this can translate into indiscriminate acceptance of results. UGRI students become more discerning by learning how databases are built, the kinds of information they provide, and their limitations. For example, the students learn that the results of BLAST searches for their DNA fragment are only sequence-based hypotheses about its function rather than an absolute answer. As students progress, they use more advanced tools for data interpretation such as the Pfam database [10] and IMG [11] to develop sequence-based hypotheses about the metabolism of A. degensii [12] (Text S1).

By producing their own sequence data, students acquire both theoretical understanding and research skills. They can evaluate their experimental performance and see how their laboratory technique affects the sequencing gel, which in turn can influence their bioinformatics result. This self-generated feedback motivates students to master techniques with a real sense of achievement. Students appreciate the opportunity to perfect their techniques by repeatedly practicing the same skill set [13,14] (Text S1), while at the same time producing a novel result: a new piece of the genome puzzle. This is in stark contrast with traditional laboratory courses in which students apply different methods each week to obtain a pre-ordained result. Moreover, LS187 students gain self-confidence by mastering theory through practice [13, 14] (Text S1): 86% of LS187 students indicated that the course made them more interested in research.

Within a research course, teamwork is essential and requires communication, accuracy, professionalism, and accountability (Text S1). To ensure that each day's sequencing is a success, students must carefully record their work, noting any mistakes or peculiarities for their colleagues. At the same time, the informal research laboratory atmosphere creates an ambience that fosters collegiality among students from a variety of different majors.

With its emphasis on a large research project, tractable only through collaboration, the courses in the UGRI form a learning community that transcends departmental and course boundaries. Most LS187 students were introduced to the project through their earlier participation in the only prerequisite course, “Introduction to Molecular Biology” (LS3). Each quarter, LS187 students make a presentation about the course to LS3 students, and all students can follow the sequencing progress and latest news about the UGRI via its public website [5].

Unlike the traditional role of students as knowledge consumers, UGRI undergraduates are knowledge producers. An overwhelming majority of students report that knowing their sequence data is available to other researchers on the NCBI website makes their work more interesting and motivates them to do their best (Text S1).

## From Inception to Success

The development of a program like the UGRI requires the concerted efforts of faculty, administrators, funding organizations, and biotechnology companies [2,15]. Partnership with biotechnology companies is essential and synergistic; they provide state-of-the art equipment and reagents—the raw material for education innovation—and in return, the next generation of researchers becomes familiar with their products. Faculty in associated courses must be willing to work together, try new things, and adapt their courses and their teaching styles. Furthermore, it's a different kind of teaching with research, and one that emphasizes understanding of the process of science, rather than focusing on memorizing the facts—its products.

The early success of this innovative program is revealed at several levels. Since the course started in the fall of 2003, nearly 2 Mb of sequence have been generated and assembled into contigs, with annotation proceeding at an accelerating pace. A partnership with the Department of Energy's Joint Genome Institute has deepened the collaborative scope of the program. Forthcoming scientific publication will document this productivity. Student interest continues to grow: the course is at capacity with a wait list, and so far, over 5000 students from eight different courses have participated. Student evaluations in all these courses laud the experiences. Additionally, we have developed assessment methods to monitor the impact of the UGRI on an ongoing basis [13,14]. We plan to track students over the next 5 years to document the downstream effects of the UGRI on academic progress and career choice.

## Conclusions and Outlook

The UGRI demonstrates how research universities can answer the call to take a leadership role in developing and evaluating innovative undergraduate education programs [16]. The successful expansion of the associated courses network to liberal arts schools [17] and smaller universities suggests that the strategy of creating a research project specifically for undergraduates could readily be adopted by all types of undergraduate institutions, singly or through collaborations (equipment permitting). Protocol S1 provides a detailed guide to creating and managing the hub course for a microbial genome sequencing project, however, we believe the UGRI template could be applied to a whole range of projects—such as sequencing organelle genomes, expressed sequence tags, or environmental samples. The scope and goals will ideally fit the environment and the student and faculty interests at each institution.

A genomics-based research project puts students side-by-side, carrying out the same techniques repeatedly, working toward a common goal. This parallel approach to undergraduate research has the corollary benefit of reducing mentoring effort; all students learn the same sets of techniques (experimental, computational, and analytical), which they have the opportunity to master thanks to repeated practice under the guidance of their peers. As the UGRI demonstrates, genome sequencing provides a platform to allow large numbers of students to participate in research and to connect a network of concepts from across the life sciences disciplines.

## Supporting Information

Figure S1 Flow of Experimental Steps among the Associated Courses and LS187 (46 KB PNG).

Protocol S1 LS187 Course Materials and Methods (806 KB DOC).

Table S1 LS187 and UGRI Associated Courses at UCLA (23 KB DOC).

Text S1 Student Description of Annotation Experience and Results of Student Exit Surveys (87 KB DOC).
