# Supplementary figures and images for: The Undergraduate Genomics Research Initiative
Source: PLoS Biol. 2007 May 15;5(5):e141. doi: 10.1371/journal.pbio.0050141 (PMC1868073; doi:10.1371/journal.pbio.0050141)

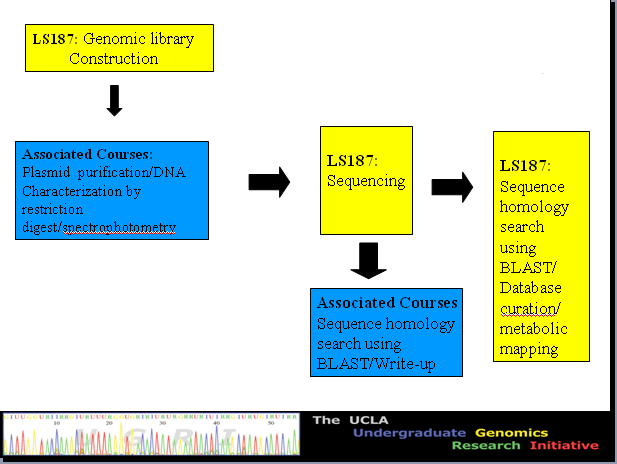

Supplement: Figure S1 — (46 KB PNG). [file pbio.0050141.sg001.png]
