# Supplementary material for: The Undergraduate Genomics Research Initiative
Source: PLoS Biol. 2007 May 15;5(5):e141. doi: 10.1371/journal.pbio.0050141 (PMC1868073; doi:10.1371/journal.pbio.0050141)

**Protocol S1: Curriculum Materials and Methods**

**Index**

I. [Student Recruitment and Orientation](#Student_Recruitment)

II. [DNA Sequencing and Analysis](#DNA_Sequencing_and_Analysis)

A. [Genomic DNA Preparation](#Genomic_DNA)

B. [Library Expansion](#Library_Expansion)

[Ligation](#Ligation)

[Transformation](#Transformation)

[Inoculation](#Inoculation)

[Glycerol Stocks](#Glycerol_Stocks)

[Obtaining Bacterial Cell Pellets](#Cell_Pellets)

C. [Isolating and Characterizing DNA From the Genomic Library](#Isolating_and_Characterizing_DNA)

[Plasmid Prep](#Plasmid_Prep)

[Enzymatic Digest](#Enzymatic_Digest)

[Gel Electrophoresis](#Gel_Electrophoresis)

[Spectrophotometry](#Spectrophotometry) (DNA Concentration and Purity)

D. [DNA Sequencing and Analysis](#DNA_Sequencing_and_Analysis)

[Acrylamide Gel Slab Preparation](#Acrylamide_Gel_Slab)

[DNA Sequencing Reaction](#DNA_Sequencing_Reaction)

[PCR-based DNA Amplification](#PCR_based_DNA_Amplification)

[Loading DNA Samples](#Loading_DNA_Samples) into Gel Slab Using LI-COR 4200 Sequencer

[Gel Analysis](#Gel_Analysis)

[Bioinformatics](#Bioinformatics) - BLAST

E. [Data Quality Control and Archiving](#Data_Quality_Control)

[Cross-Checking Analyzed Gels](#Cross_Checking_Analysed_Gels)

[Reviewing BLAST Results](#Reviewing_BLAST_Results)

[Archiving Project Results](#Archiving_Project_Results)

III. Sample Logs

[Weekly Duty Roster](#Duty_Roster)

[PCR-based DNA Amplification Log](#PCR_Log)

[Clone Log](#Clone_Log)

[Gel Sequencing Log](#Gel_Sequencing_Log)

[Setting Lanes Manually – Sample Gel](#Lane_Setting)

[BLAST Log](#BLAST_Log)

[BLAST Error Log](#BLAST_Error_Log)

[Archived BLAST Log](#Archived_BLAST_Log)

**I.** **Student Recruitment and Orientation**

Interested undergraduates must have completed an introductory course in molecular biology (LS3). To apply for the course, students complete a questionnaire as to academic background, lab experience, and motivation for enrolling in the UGRI. Enrollment for course is capped at 25 students, and for the advanced series (B and C courses) at 10 students.

Student researchers are required to conduct research for six hours per week; each shift must be at least two hours in length. Approximately half of the student researchers work two three-hour shifts per week and the rest work in three two-hour shifts per week. The students are assigned duties (see [sample roster](#Duty_Roster)). Team composition and assigned tasks are changed weekly so that every student researcher completes each lab activity at least twice with a different teammate. This ensures adequate cross-training and improved collaboration among the students.

The first week of the course is devoted to orienting the students to the lab equipment and conducting mock exercises to master wet lab skills necessary for genomic sequencing. The core protocols practiced include assembling gel slabs for the DNA sequencer, performing a sequencing reaction and loading DNA samples into sequencing gels (LI-COR 4200 DNA analyzer) (see [DNA Sequencing and Analysis](#DNA_Sequencing_and_Analysis) for overview of these lab activities). The most important technique practiced during the first week is mastering the proper use of the pipetteman. Experience has shown that students lack proficiency with this tool despite most having had occasional use of a pipetteman in other science courses. Accordingly, every student is assigned a volume to pipet. They must set the pipetteman and transfer a discrete volume of water which is then weighed to check precision.

**II. DNA Sequencing and Analysis**

**A.** **Genomic DNA Preparation**

Genomic DNA was isolated (using the method of Lauerer et al., Appl. Microbiol. 8, 100-105, 1986) from cell pellets of *Ammonifex degensii* that had been repeatedly freeze-thawed. The DNA was size fractionated on an agarose gel. These steps were carried out by the instructor or by advanced students under the supervision of the instructor.

**B.**  **Library Expansion**

Library expansion by the students generally involves teams of four students working in a serial fashion. Each team expands 40 to 50 clones per shift. Library expansion follows strict aseptic conditions to avoid contamination; all pipet tips, test tubes and other materials autoclaved prior to use. Stock Kanamycin (50 mg/ml) plates are prepared and stored at -20 degrees Celsius.

***Li******gation***

LBKan (50 μg/ml) plates are removed and allowed to dry prior to use. DNA fragments to be ligated are placed on ice. Ligation is completed using Zero Blunt® TOPO® PCR Cloning Kit (Invitrogen, Carlsbad, California). Reagents for this kit for a single reaction are:

4.0 μl of *A. degensii* DNA

1.0 μl NaCl solution

1.0 μl TOPO vector

All components are gently swirled using an autoclaved pipette tip. The ligation product is incubated for 30 minutes at room temperature and then placed on ice.

***Transformation***

One vial of One Shot® Chemically Competent E. coli cells (Invitrogen) are defrosted for 5-8 minutes and then placed on ice. A 5 μl aliquot of the ligation product is added to thawed cells and incubated on ice for 10 minutes. The mixture is subsequently placed at 42 degrees Celsius for precisely 45 seconds, without shaking the mixture, and then promptly placed in ice for two minutes. A 250 μl aliquot of SOC broth (warmed to room temperature or 37 degrees Celsius) is added to the cells without mixing. The vial is capped and sealed with paraffin and incubated in a shaker (37 degree Celsius, 200 rpm) for one hour. Students plate out 150-200 μl of bacterial culture onto LBKan (50 μg/ml) plates and incubate them overnight at 37 degrees Celsius. Plates are removed the following day, wrapped in paraffin, and stored at 4 degrees Celsius until inoculation.

***Inoculation***

Strict aseptic techniques are observed during inoculation. Bench top and gloved hands are sterilized with 70% EtOH solution. Pipet tips and inoculation test tubes are quickly flamed during the procedure. All work is conducted near an open flame. Bacterial plates are labeled into four quadrants. The edges of selected colonies are picked using an autoclaved pipet tip taking care not to touch other colonies on the plate. The pipet tip with the selected bacterial colony is then deposited into a sterile tube containing LB Kan medium. The mouth of the tube is quickly flamed and capped. This process is repeated for 40 colonies. Cultures are incubated in a shaker (37 degrees Celsius, 200 rpm) overnight. Exhausted plates are discarded following biohazard disposal protocols.

***Glycerol Stocks***

Glycerol stocks of each clone are prepared to ensure a viable stock of clones are available for future use. All clones are therefore available for subsequent sub-cloning processes, to reconstruct a particular gene of interest, or to share with other researchers. This task is usually assigned to 2-3 students with tasks performed in a serial manner (i.e., one student labels cryogenic vials, another adds glycerol, and so on) following strict aseptic techniques. Each shift processes 40 clones into glycerol stocks.

Bench areas are sterilized with 70% EtOH solution and all work is conducted near an open flame. Pipet tips and cryogenic vials are autoclaved prior to use. A 150 μl volume of sterile glycerol is added to each autoclaved cryogenic vial and then promptly capped. An 850 μl volume of bacterial culture, obtained from the prior day’s tubes of cultured bacteria (see [Inoculation](#Inoculation)), is added to each vial labeled with the appropriate clone number. All test tubes are flamed prior to capping. Capped vials are quickly vortexed and then set upon dry ice for quick freeze. All glycerol stocks were placed in -70 degree freezer for long-term storage. The physical location of stored clones in the freezer were logged by the assembly team.

***Obtaining Bacterial Cell Pellets***

Cell pellets are collected so that associated classes can extract recombinant plasmids from *E. coli* (see [Plasmid Prep](#Plasmid_Prep)). Two student researchers conduct this task in a serial manner (i.e., one pipets cultures into labeled microcentrifuge tubes while the other centrifuges the mixture). Each team processes 80 cell pellets during each shift. Two cell pellets for each clone are produced.

Bench areas are sterilized with 70% EtOH, and all work is conducted near an open flame. Pipet tips and microcentrifuge tubes are autoclaved prior to use. All culture spills are immediately cleaned with bleach, 70% EtOH, and water. A 1.5 ml volume of bacterial culture, obtained from the prior day’s culture of bacteria (see [Inoculation](#Inoculation)), is pipetted into two microcentrifuge tubes, each labeled with the appropriate clone number. The cultures are centrifuged for 5 minutes at 14,000 rpm. After decanting the supernatant, the pellet is stored at -70 degree Celsius.

**C.** **Isolating and Characterizing DNA From the Genomic Library**

Minipreps are completed as a lab assignment for undergraduate students enrolled in Associated Courses. Each student receives one clone. LS187 student researchers also perform these tasks as required.

***Plasmid Prep***

Plasmid DNA is extracted using the Eppendorf® FastPlasmid™ Mini Kit (Eppendorf) or with the QiaPrep® Spin Mini kit (Qiagen), using manufacturers’ protocols. Alternatively, standard alkaline lysis protocols are used.

***Enzym******atic Digest***

DNA samples are digested with *EcoR I* taking advantage of the restriction site flanking the region where the insert was incorporated into the pCR®-Blunt II TOPO® plasmid (Invitrogen). Thus, *EcoR I* digestion releases the *A. degensii* DNA insert from the plasmid. The reagents for a single reaction are:

10.0 μl dd H_2_O

2.0 μl 10X Buffer H

2.0 μl 1X BSA

5.0 μl of [plasmid prep](#Plasmid_Prep) DNA

1.0 μl EcoR I (10U/ul)

The mixture is quick vortexed followed with a quick centrifuge spin. The digest was incubated in a 37 degree Celsius water bath for one hour. After incubation, samples are placed on ice followed by a quick centrifuge spin to remove any condensation. A 3.3 μl volume of 6X loading buffer is added into each sample followed with gentle mixing via a pipetteman. Samples are either stored in -20 degree Celsius freezer for several days or examined by gel electrophoresis on the same day.

***Gel Electrophoresis***

This step uses a 1.0% agarose gel using 1X TAE or 1xTBE with ethidium bromide (1.0 μl per 1.0 g agarose). The running buffer used is same as that used for agarose gel preparation. Approximately 23.3 μl of digested DNA (see [Enzymatic Digest](#Enzymatic_Digest)) is added to each well along with two wells separately loaded with 5.5 μl of 100 bp and 1 kb ladders, respectively. Gels are run for 45 minutes at 120V for one hour until visible separation of colored bands. Slabs are photographed under UV light and attached to a log matching clone numbers with wells (sample not provided).

***Spectrophotometry (DNA Concentration and Purity)***

DNA concentration and purity is obtained via spectral analysis. A 1:100 dilution of plasmid DNA was made in water and measured for A260, A260/280, and converted to concentration (μg/μl). Students log readings for each measured clone in Clone Log (see [sample](#Clone_Log)).

**D*.*** **DNA Sequencing and Analysis**

***Acrylamide Gel Slab Preparation***

Each day, a team of two student researchers prepare the gel slab. The assembly unit is thoroughly cleaned with 2% NP detergent and scrubbed with non-abrasive brushes. Glass plates are rinsed with distilled water followed with 70% EtOH and wiped down using Kimwipes. The gel matrix is comprised of 30.0 μl of 5.5% KB^Plus^ Gel Matrix (acrylamide; LI-COR) allowed to warm to room temperature, mixed with 200.0 μl of 10% ammonium persulfate and 20.0 μl of TEMED. Bubbles are removed with bubble hooks. A 0.2mm casting comb is inserted prior to polymerization. The gel slab polymerizes in ~1.25 hours.

After removal of 0.2 mm casting comb, the well is rinsed with 22 gauge needle using a solution of 0.8X TBE (LI-COR KB^Plus^) running buffer to ensure proper well configuration and to remove residual urea. Finally, a 64-sharktooth comb is inserted into the well at eye level of running buffer in upper reservoir of LI-COR 4200 sequencer. Upper and lower reservoirs are filled with freshly prepared 0.8X TBE (KB^Plus^) running buffer. Students enter any abnormalities (e.g., bubbles, obstructions from lint, etc.) onto a Gel Sequencing Log (see [sample](#Gel_Sequencing_Log)) for the day’s run.

***DNA Sequencing Reaction***

The course used either the Sequenase™ DNA Sequencing Kit (USB, Cleveland, Ohio) or the SequiTherm EXCEL™ II DNA Sequencing Kit (Epicentre® Biotechnologies). Primers used in both kits are light and temperature sensitive and accordingly are wrapped in aluminum foil and stored on ice during use and stored during non-use at 4 degrees Celsius. A total of 16 DNA sequencing reactions are completed daily by two student researchers (this corresponds to the 64 available wells in the gel slab). Each researcher logs results in a PCR Log (see sample).

USB’s Sequenase™ DNA Sequencing Kit – Bidirectional Sequencing:

The following reagents are thawed for 20 minutes prior to use: DNA, Thermo Sequenase reaction buffer, ddNTP master mix, ddNTPS (ddATP, ddCTP, ddGTP, and ddTTP), SP6 (forward IRD 700nm primer), T7 (reverse IRD 800 nm primer). .

Epicentre® SequiTherm EXCEL™ Kit:

Reagents used for a single reaction are:

1.5 μl of IRD700 forward primer (1.0 pmol/ μl)

1.5 μl IRD800 reverse primer (1.0 pmol/ μl)

7.2 μl of Sequencing Buffer

1.0 μl of EXCEL II DNA polymerase

All reagents are pipetted into a sterile microcentrifuge tube and placed on ice. A 96-well PCR plate is labeled with columns for DNA and the A, C, G and T termination reactions and kept on ice throughout the reaction. A 2.0 μl volume of each termination mix (ddATP, ddCTP, etc.) is added to each corresponding well. A 1.2 μl volume of the master mix and 8.8 μl volume of DNA is mixed into the DNA well; after mixing, a 4.0 μl aliquot of the resulting mixture is pipetted into each termination well taking care to change tips between each aliquot.

***PCR-based DNA Amplification***

Single stranded *A. degensii* DNA inserts are amplified using a PCR-based (Sanger method) in a thermocycler (MyCycler, Bio-Rad or Apollo, Continental lab Products). Denaturing occurs at 92 degrees Celsius, annealing at 50 degrees Celsius, extension at 72 degrees Celsius and holding at 4 degrees Celsius. A 3.0 μl volume of IR2 stop solution (LI-COR) is added into each reaction at completion. Each student researcher logs results for each sequenced clone (see [sample log](#PCR_Log)).

***Loading DNA Samples into Gel Slab using LI-COR 4200 Sequencer***

This task is accomplished by two student researchers. Sequenced DNA is denatured at 92 degrees Celsius for three minutes prior to loading. One student pipets 1.0 to 2.0 μl of DNA samples (i.e., A, C, G, and T of each clone) into successive wells taking care to avoid deformed wells or those lanes containing bubbles or other contaminants as indicated by the Gel Log. Errors or improper technique (e.g., spillover from well overloading) are logged by the other student in the Gel Sequencing Log (see [sample log](#Gel_Sequencing_Log)).

***Gel Analysis***

The LI-COR 4200 can sequence bi-directionally. Students analyze gels using LI-COR’s e-Seq program (version 2.0) which generates chromatogram (trace) files for the forward (IRD 700nm) and reverse (IRD 800nm) sequencing. Lanes are assigned manually (see [example screen shot](#Lane_Setting)) to account for loading abnormalities (e.g., skipped lanes, bad loads, overflow contamination) set forth in the Gel Sequencing Log (see [sample log](#Gel_Sequencing_Log)). Two students separately analyze the same gel and save their results as versions A and B, respectively. (This is done for quality control purposes – see [Cross-Checking Analyzed Gels](#Cross_Checking_Analysed_Gels).) DNA Star’s Seq-man is used to develop contigs of the forward and reverse sequences.

***Bioinformatics - BLAST***

Two students are separately assigned the same eight trace files to BLAST against NCBI’s database. (This is done for quality control purposes – see [Reviewing BLAST Results](#Reviewing_BLAST_Results)). Each researcher generates FASTA files from each chromatogram, documents the top ten BLAST hits, and logs key details of the top BLAST hit (e.g., protein hit, species, e-value, identities, etc.) (see [sample log](#BLAST_Log)). All files are prepared and formatted pursuant to NCBI protocols.

**E.** **Data Quality Control and Archiving**

***Cross-Checking Analyzed Gels***

This is assigned to an advanced LS 187 student researcher (enrolled in LS187B or C) who is competent in the methodology of gel analysis but who has not participated in the original analysis of the gel in question. The checker evaluates versions A and B of the same gel (see [Gel Analysis](#Gel_Analysis)), which was analyzed separately by two students, and selects the version that contains the least number of ambiguities and gaps. Final results are reviewed by the lab instructor. The selected gel is used to produce the trace files submitted to NCBI.

***Reviewing BLAST Results***

This is assigned to an advanced LS 187 student researcher who is competent in the methodology of BLAST but who has not completed the original BLAST analysis. The checker verifies that (i) files are named, formatted and logged correctly (pursuant to NCBI protocols), (ii) FASTA files generated by the trace file is properly edited (e.g., vector sequence and trailing ambiguities omitted from BLAST nucleotide sequence), and (iii) that BLAST results represent the best hit (e.g., check e-values, identities, species hit, etc.) This information is then logged into a BLAST error log (see [sample](#BLAST_Error_Log)). The lab instructor then reviews the error report with the student researchers; common problems are discussed during the weekly all-hands lab meeting.

***Archiving Project Results***

This is one of the final steps in preparing the data for submission to NCBI database. An advanced LS 187 student researcher (sometimes two) handles this task with lab instructor supervision. The best BLAST data for the same clone prepared separately by two students (see [Bioinformatics](#Bioinformatics)) are used for NCBI submission. Data may be selected from the A version, B version, or a mixture of both versions. The best BLAST data (e.g., FASTA files and BLAST output, see [sample](#BLAST_Log)) are merged and linked together into the lab’s in-house database (see [sample](#Archived_BLAST_Log)). Final results are reviewed by the lab instructor.

**Sample Weekly Duty Roster** (link to [Narrative](#Assigning_Duties))

**Key to Columns**:

**Sequencing**. Daily. Individual researchers are assigned to complete DNA sequencing reactions (link to [discussion](#DNA_Sequencing_Reaction)).

**Gel**. Daily. Two student researchers clean and prepare an acrylamide gel slab for the day's lab (link to [discussion](#Acrylamide_Gel_Slab)).

**Lab**. Daily. Library expansion by the students generally involves teams of four students working in a serial fashion. Each team would expand 40 to 50 clones per shift. One team performs autoclaving (link to [discussion](#Autoclaving)) while another performs library expansion (link to [discussion](#Library_Expansion)).

**Analyze A, B and AB**. Daily. An advanced student researcher evaluates two versions of the same gel that was analyzed separately by two students (link to [discussion](#A_B_Gels)). The checker selects that version having the fewest ambiguities (link to [discussion](#Cross_Checking_Analysed_Gels)).

**SeqMan**. Daily. An advanced student researcher prepares contigs of the forward and reverse sequences and assigns that are eventually BLASTed against NCBI’s database (link to [discussion](#SeqMan))

**BLAST/Link**. Daily. An advanced student researcher selects the best BLAST data (e.g., FASTA files and BLAST results) and merges and links them together into the lab’s in-house database (link to [discussion](#Archiving_Project_Results)).


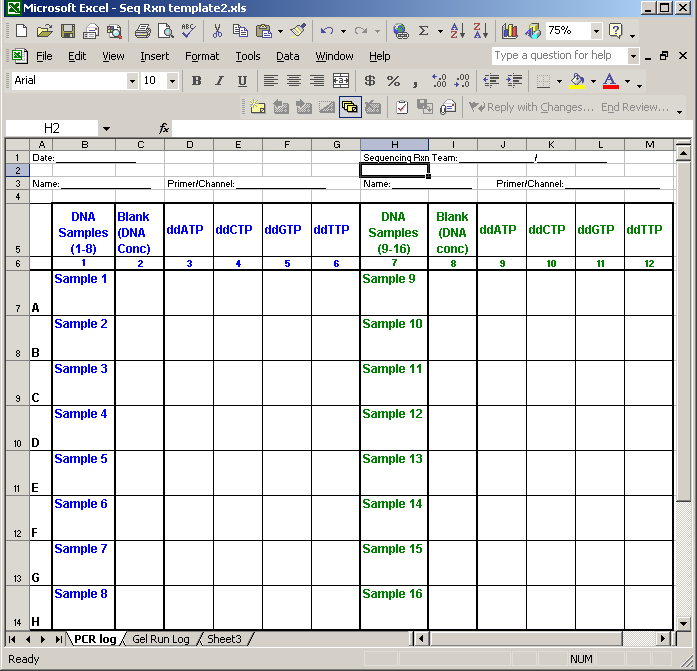
**Sampl****e PCR-based DNA Amplification Log** (link to [Narrative](#PCR_based_DNA_Amplification))

**Sample C****lone Log** (link to [Narrative](#Spectrophotometry))


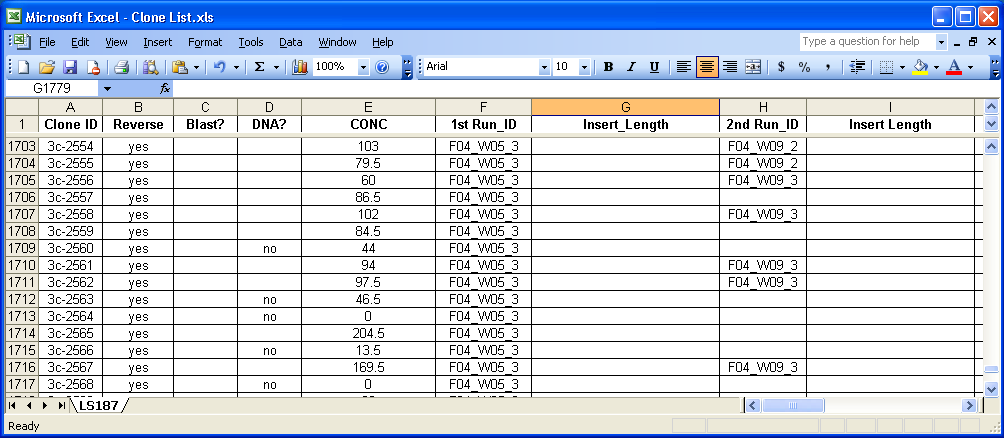


**Sample Gel Sequencing Log** (link to [Narrative](#Gel_Loading))

**Setting Lanes Manually – Sample Gel** (link to [Narrative](#Gel_Analysis))


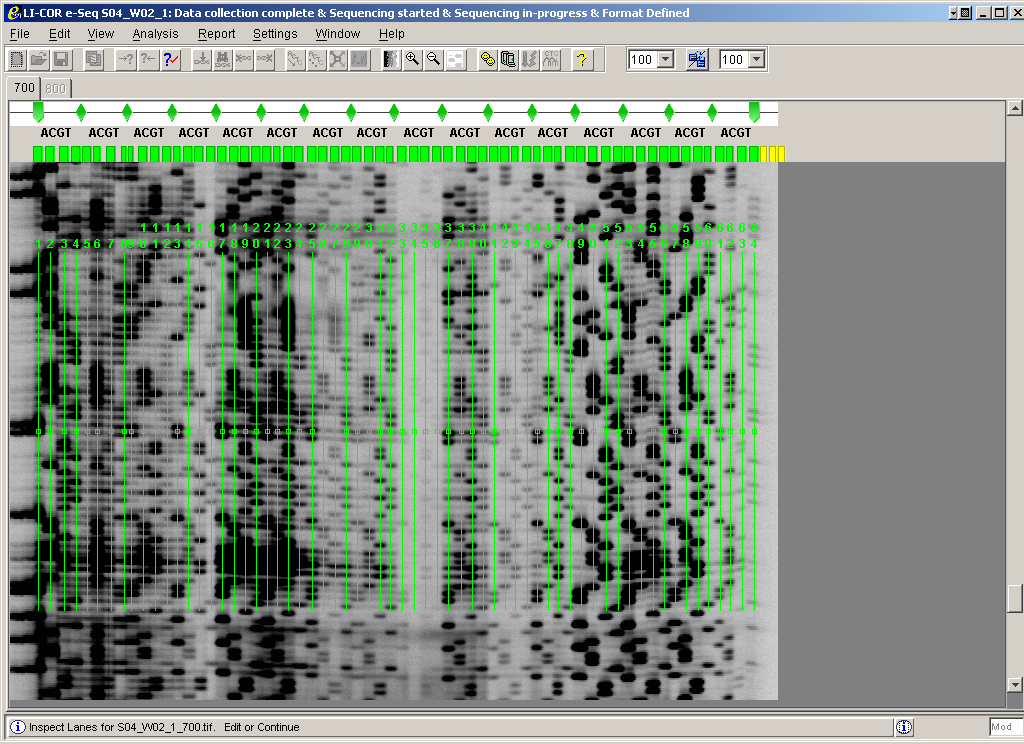


**Sample BLAST Log** (link to [Narrative](#Bioinformatics))


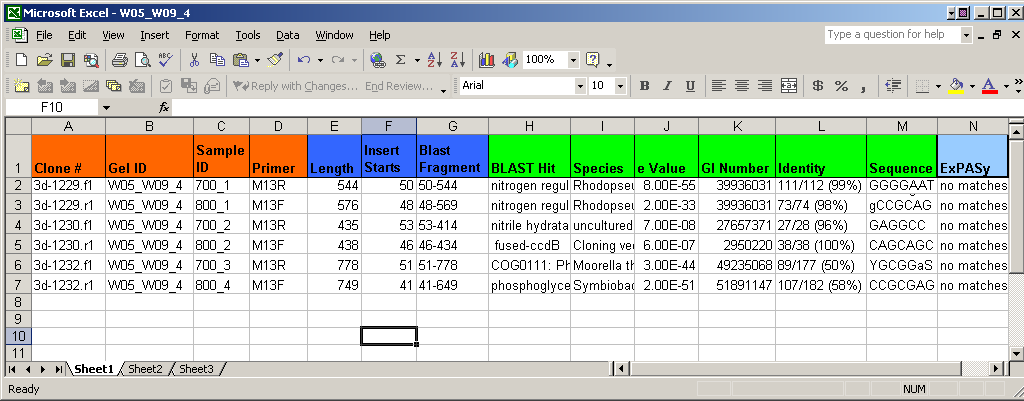


**Sample BLAST Error Log** (link to [Narrative](#BLAST_Review))


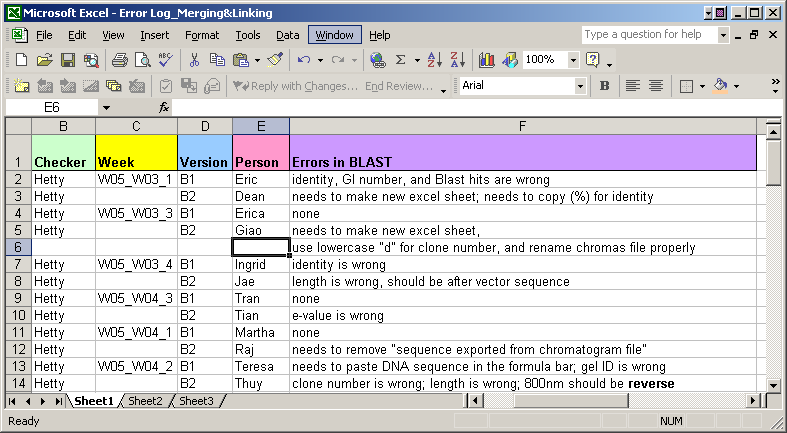


**Sample Archived BLAST Log** (link to [Narrative](#Archiving_Project_Results))


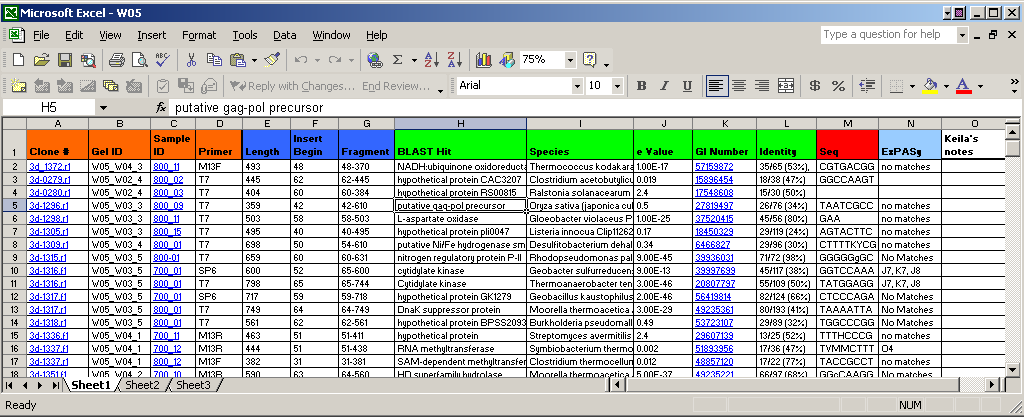

Supplement: Protocol S1 — (806 KB DOC). [file pbio.0050141.sd001.doc]
