# Supplementary material for: The Undergraduate Genomics Research Initiative
Source: PLoS Biol. 2007 May 15;5(5):e141. doi: 10.1371/journal.pbio.0050141 (PMC1868073; doi:10.1371/journal.pbio.0050141)
Supplement: Table S1 — (23 KB DOC). [file pbio.0050141.st001.doc]

**Table S1: LS187 and UGRI Associated Courses at UCLA**

**1. LS187 Principles and Practices of Genomics Research**

Enrollment: 25 students/10 week quarter

Typical Student: Sophomore to Senior; the only prerequisite is Life Sciences 3, “Introduction to Molecular Biology” The course is an elective but fulfills the majors’ research requirement for some UCLA departments (e.g. Molecular, Cellular and Developmental Biology, Microbiology, Immunology and Molecular Genetics).

**2. Associated Courses at UCLA**:

**A. General Education 71, Biotechnology and Society** Enrollment: 140 students annually

Typical Student: Freshman, nonlife-sciences major

Catalog Description: Lecture, three hours; discussion, two hours; laboratory, two hours. Exploration of methods, applications, and implications of biotechnology and of ethical, social, and political implications as well as biological underpinnings. Letter grading.

**B. Life Sciences 3, Introduction to Molecular Biology**

Enrollment: 1700 students annually (quarterly enrollment varies)

Typical Student: Sophomore and Juniors intending to major in the life sciences.

Catalog Description: Lecture, three hours; discussion/laboratory, three hours (alternate weeks). Enforced requisites: course 2 or former course 2W, Chemistry 14C or 30A. Introduction to basic principles of biochemistry and molecular biology. Letter grading.

**C. Microbiology, Immunology and Molecular Genetics 120, Advanced Techniques in Microbiology**

Enrollment: 48 students/10 week quarter

Typical Student: Senior level, Microbiology, Immunology and Molecular Genetics major.

Catalog Description: Lecture, one hour; laboratory, six hours. Requisite: course 101L or 102L, with a grade of C or better. Introduction to current recombinant techniques. Experiments include PCR, cloning, and other recombinant techniques. DNA binding recombinant protein is purified from "Escherichia coli" and its ability to bind to DNA studied using gelshift assay. Introduction to protein/protein interaction using yeast two-hybrid system and to tissue culture techniques and transfection and expression of genes for human light and heavy chain antibody. Concurrently scheduled with course C220. Letter grading.
