# Supplementary material for: The Undergraduate Genomics Research Initiative
Source: PLoS Biol. 2007 May 15;5(5):e141. doi: 10.1371/journal.pbio.0050141 (PMC1868073; doi:10.1371/journal.pbio.0050141)
Supplement: Text S1 — (87 KB DOC). [file pbio.0050141.sd002.doc]

**Text S1**

**A. Student description of *A. degensii* annotation experience**

One LS187C student described his experience in making some preliminary annotations of the *A. degensii* genome sequence: “Amazingly, I was able to tentatively describe *A. degensii’s* carbon fixation pathway by starting with a handful of data: sequenced gene fragments from *A. degensii’s* genome; related BLASTx protein hits and their associated organisms; its phylogeny; and its biotope…. Using IMG’s curated database, I found the separate orthologous gene clusters within *M. thermoacetica* that contained the three diagnostic W-L pathway enzymes found in our in-house database. I then determined the enzymes that flanked each diagnostic enzyme to see whether they too were represented in our in-house database. Many such associated enzymes were common redox enzymes (e.g., 4Fe-4S ferredoxins; hydrogenases) that are sometimes associated with different pathways. So, I completed recriprocal BLASTx to verify that the in-house protein in question had significant homology to that found in the orthologous gene cluster. I confirmed, for example, that not all of the 4Fe-4S ferredoxins and hydrogenases found in our database had significant homology to those found in *M. thermoacetica’s* orthologous gene clusters associated with the W-L pathway (that is, some were associated with other metabolic pathways, not carbon fixation). Ultimately, I found enough correlation between associated proteins found in our database and those found in *M. thermoacetica’s* W-L pathway gene clusters to tentatively conclude that *A. degensii* is an acetogen.”

**B. Excerpts from LS187A Student Exit Surveys, 2004-2005 and 2005-2006 academic years. The responses are grouped thematically, with the question prompt given in italics.**

**1. Research and student learning**

*Did the importance of what you were doing, sequencing a microbial genome and depositing your data in a worldwide databank, influence your level of motivation or interest in your LS187 work?*

*Did this class increase your interest in doing research?*

“The class gives an idea of how interesting research can be and also how teamwork influences the outcome of research. I now consider doing research after graduation.”

“I have always been highly interested in research; this class just further solidified my passion for research.”

“I was motivated extra in this course, because I felt I was contributing to a worldwide database. My work seems significant and thus increased my interest in my work.”

“The fact that our data was being contributed to the worldwide databank motivated me to produce valuable results since the results were being actually utilized. In contrast to a regular lab class, it usually does not matter if you do not obtain the correct results since one experiment does not depend on the other. In LS187A, I not only had to figure out why the experiment did not work, but I also had to redo the experiment since we had a real goal to meet.”

I learned so many skills in this class: lab techniques, use appropriate terms to “sound” more like a scientist, and how to think as a scientist. This class was very valuable and enhanced my desire to pursue research.

“I really enjoyed taking this class because I was able to learn a lot of research techniques.”

“In other lab courses, I read a manual and perform an experiment that will later be discarded after obtaining the results, which should always be consistent. However, in this lab, the outcomes are different because this project hasn’t been performed by previous researchers. Therefore, each different outcome enhanced the learning of the researcher because he/she will need to think about why the result occurred.”

“This class makes you think how researchers work in the sense that if something goes wrong you have to try and fix it. It makes you also think of how to improve things to be more efficient.”

“In traditional lab courses, I feel like I am just following a protocol and doing mindless work. In these courses, for the most part, the results of the experiment do not matter. In this course, I had to think more on my feet, and I was more responsible for the quality of my work.”

“This class is a great learning experience. Thinking analytically about each step of the procedures is much more educational than mindlessly following procedures from a book.”

“Because the results of our work were uncertain in contrast to “canned labs”, it really forced me to stay focused and really make an effort to understand techniques utilized in lab.”

“Research done in the style of this course is a much better learning experience. I felt I learned not only concepts but also how a lab worked, the process of scientific discovery, and problem solving”

“I mean why waste money and effort on those predictable lab courses where the results are not counted?”

“Knowing that what we were doing was not merely another experiment to occupy our time, but a part of a more important and “realistic” project influenced my want to be more efficient and produce more work of better quality.”

“It is an excellent class to take especially if one has never done research before. It is a good place to start.”

“This is the best biology based course I have ever taken and the most fun I‘ve had with biology in a long time. In the end and I even enjoyed doing the BLAST homework and got really excited when I got a low e-value (3x10^-114^) for a *Moorella* match.”

“I think, above all else, undergrads running our own research project builds more camaraderie. It seems to simulate what research is really like. While doing canned labs, I thought research was so boring and dull. Now I know what the atmosphere is really like in a lab and so I don’t feel deterred from doing research in the future.”

“I really did not have any interest in genomics and researching the microbial genome before this class. But after going through this class, I enjoy what I was doing and understood a purpose to the lab work. I wish all classes were like this.”

“I enjoyed the experience because unlike, say, the experiments in Chemistry 30BL, the results are unpredictable and require analysis themselves. I also like the fact that the results count for not just grades, but actual research data.”

“Usually I forget what I do in regular lab classes soon after I turn in the lab report. This class, on the other hand, allowed me to have a stronger grasp on lab techniques and concepts.”

“‘Canned’ experiments allow you to forget about the experiment after you’re done with it, while repetitively doing it will forever engrain it into one’s mind.”

“It is so much better to apply techniques learned in lab than to just read about them in a textbook. I feel like I’ve picked up skills from this class that I will definitely be using in the future.”

“I found this class to be leaps and bounds better than most ‘canned’ labs. I was less stressed out and believe I actually learned more due to decreased anxiety.”

“First, I want to say that some techniques that I met in this lab, I couldn’t actually get the opportunity to perform in previous labs that I worked. Also, techniques combined Molecular Biology, Bioinformatics (such as BLAST…), and Microbiology (Inoculation…). This was way better than “canned” experiment each week scenario. Since in LS187, you get to master one topic and then you can do the same in B&C. The fact that outcomes were different made me even more familiar with what might go wrong, why and what to expect. We also did get to work on more than one topic i.e. we rotated lab techniques every 5 weeks which was also helpful.”

“I liked the independence undergraduates can exhibit through this course. This class is like a puzzle rather than just a textbook with stuff we need to memorize and regurgitate. Years later, I won’t remember a lot of facts I had to memorize through my lectures classes, but I will remember how to make a gel, read a gel, and troubleshoot problems.”

“I believe the more hands-on approach is far more valuable as a learning device. You get the knowledge visually plus you are able to apply this knowledge in a working background.”

“Definitely conducting different experiments with unexpected outcomes improves one’s problem-solving techniques. Each experiment itself contains unexpected outcome so one has to learn to solve problems as they occur. After taking this course, my laboratory skills shave definitely improved from all these different tasks because repetition of lab work has helped me become adroit at my tasks.”

“In contrast to the cookie-cutter procedures of other lab courses, everyone in this class can modify/improve the procedures in this constantly changing lab to suit new needs. This class allows for troubleshooting and problem solving since procedures may not be available for all things.”

“I have hated the over-enforced structure of previous labs; simply copying instructions and going through the motions. This class was for more dynamic and engendered a deeper and broader lab experience.”

“I really did not have any interest in genomes and researching of microbial genomes before this class. But after going through this class, I enjoyed what I was doing and understand a purpose to the lab work. I wish all classes were like this.”

“There are many critical path items where a small screw up results in hours of lost time. Finally mastering (and I use that term lightly…) the techniques made me feel sort of like a scientist rather than a student. Much more motivation to learn and explore, particularly given what’s at stake.”

“It definitely sparked an interest in me to learn more about genomics.”

“I like the unpredictability of 187 and the independence. It would be super awesome if we had 20 sequencers and everyone did their own.”

“It seems to simulate what research is really like. While doing boring, canned labs, I thought research was so boring and dull. Now I know what the atmosphere is really like in a lab, and so I don’t feel negative about doing research in the future.”

“I think it influenced my interest a lot. In many of the other classes I have taken, I feel the labs we do have no real meaning (that is, it’s not really important or even relevant to what we’re learning). But knowing that our work is important, and goes into a national database give me more motivation to do well.”

“Initially, the idea of doing meaningful real-world impacting research was what drove me; it still uses this idea to spark other people’s interest. Yet as we got deeper into the quarter, I became more familiar with my tasks and more comfortable in the environment and develop a greater understanding of what we were doing. It became the lab work that kept me driven and interested.”

“This project gives a good opportunity for undergraduates to experience of running a research project. Clearly, it is also more exciting to do this sort of things than to only performing experiments whose results have been widely known for 50 years.”

“I believe the LS187 format for labs will revolutionize the ways labs are run. Comparing my past labs to this, the students are more excited and interested in the subject. They actually want to know the material and understand what’s going on.”

“I think this course is valuable. In normal lab classes, we perform experiments that are predictable, and we do them just in order to get through them. In this lab, I feel like I’m actually accomplishing something.”

“Before taking this class, I was expecting just another lab course where you do useless things such as learn how to use the manual pipette. During the middle of the course, I felt that the research can in fact, improve the environment and impact the world**.** I felt more motivated to work (e.g. such as staying longer lab hours to finish loading).”

**2. Value of lab meeting**

*Please comment on the value of the lab meetings*

“It is very valuable since we cannot meet everybody in the lab. The meeting gives opportunity for everybody to discuss what’s happening in the lab and when the people come up with better methods, they can teach other people”

“It was informative in that I could learn from others’ mistakes.”

“It helps because people share their problems in lab, so we can learn from each other’s mistakes.”

“It was very valuable to learn about the problems my classmates were having, so that we could be more productive in the lab.”

“Essential! We got issues off our chest, and even learned some key concepts/tricks to apply in lab.”

“Monday meetings are absolutely necessary, because our lab is a work in progress. Sharing innovative ideas and know-hows among the classmates improved everyone’s skills and performance.”

“It was helpful. The meeting influences our level of motivation and binds everybody together.”

“They were necessary in order to let everyone know common mistakes t o look out for and basically a good “what no to do” or”how to do better’ session.”

“I think it is very important to have the lab meeting to talk about things that had gone wrong so other people will not make the same mistake.”

“The troubleshooting portion of Mondays meetings, I believe was crucial for the success of this quarter. The troubleshooting allowed us to scrutinize and assess our problems, so we would not commit them later.”

“The concepts review and research troubleshoot were helpful to me. I was able to share lab issues with everybody else and learn from their mistakes (ex: not using borate glass plates...)

**3. Value of the theory seminars**

*Please comment on the value of the theory (Wednesday) seminars*.

“Wednesday lecture is where the magic happens.”

“The lecture helped us give an overview of the project, where the species was discovered from, who discovered it, how to deposit data in the databank, and how LS2, LS3, Biochem and O-chem have helped in this research.

“I think many students including myself are taking genetics class, and the first lecture about the Human Genome Project, and the whole genome sequence procedure actually helped us understand the differences and advantages of each method. It was also nice that we learned about Blast, Bioinformatics in depth, because my previous classes only mentioned the name Blast but never taught how it actually works.”

“I like the Wed Seminar. I feel that I learned so much about *A. degensii* and our project. I know exactly what I am doing in lab and how it is related to the whole project.”

“One of the most worthwhile experiences in this course was understanding the mechanisms of what I was doing. Either knowing exactly how the LI-COR lasers read the gels or how a particular micro-organism actually generates energy.”

“I loved the Wednesday lectures. They tied everything together. Without these, the class would have just been mundane lab work.”

“It gave context to classes that I previously thought were inapplicable to my interests”

“I think that all biological science students would benefit from a class like this because it helps give context to all the work/studying we put in over the years.”

“I was rather shocked at my level of interest in topics I normally have dismissed”

“This class allowed me to perform experiments I had read about in LS3-and never really understood-until I performed them myself.”

“It helps us to link everything together, instead of constantly thinking that they are different subjects”

“It has given me a sort of an “Insider’s edge” to what really goes on in sequencing which motivates my interests in not only this course, but also other courses.”

“This class consists of multiple of other classes. This makes me experience the real application of such courses. Before I was questioning whether such knowledge can be applied to real life. Now I am more motivated to learn them.”

“I feel that in most classes, the material never integrates anything from other classes and I am glad this class connected a lot of different ideas.”

“I can see why I took Physics 4 years ago and how it can be applied to the field of Genomics.”

“I found it extremely enriching for this course to be based upon a variety of scientific fields, bringing them together with the interest of not only doing, but understanding the work we do as we sequence the genome of *Ammonifex degensii*.”

“It helps in the way that this course brings all of the little pieces of what I learned in other courses and put them all in the picture (this project).”

“It allowed me to understand how various concepts from different fields are interrelated.”

“I found it very helpful to bring topics from other courses in the context of your work with *A. degensii*’s genome. Not only did we learn about the concepts but we also were able to apply them.”

“So often in basic science classes like physics and chemistry, I feel incredibly bored because the information doesn’t feel applicable to anything I’m interested in. It’s nice to learn that it actually is important.”

“I thought it was very useful in bringing in information from other disciplines to help understand every aspect of why we did things the way we did in our lab. It was useful comparing sequencing techniques and not just learning the biology part. I appreciated the dissection of all the aspects of the lab, not just a narrow-minded focus on one discipline.”

“I think that a multidisciplinary concept class is often better than a regular one-subject focus class! Connecting concepts from physics, chemistry, molecular biology… was way more helpful and rewarding to me. I was able to perform lab techniques and also know what was going on!”

“It was helpful to incorporate a variety of topics because it shows that scientific research isn’t only about knowing stuff in one particular field alone-a background from many subjects is necessary to get a complete picture. I felt that I got a better understanding of each concept tying different sources together.”

“I think combination of different course materials into one course definitely helps students integrate different fields of knowledge together. This way, students can understand the real application of the information instead of just the textbook definition. Moreover, it makes the class and learning experience much more interesting.”

“A macro perspective helps to “pull it all together” and bring context and relevance to these courses. I especially liked the high level--setting the bar high brings out the best.”

“ I feel this class gives one a realistic view of what performing actual research is like. The material covered in other classes was out to use and provided a multidimensional context of what other classes mean to the researcher.”

“LS187 consists of LS3, LS4, General Chemistry, and etc. This makes me experience the real application of such courses. I was questioning whether such knowledge can be applied to real life before. Now I am more motivated to learn than before because of LS187.”

**4. Value of repeating techniques**

*Please comment on the laboratory experience in LS187 relative to that in other courses. Consider that in most laboratory-based courses you perform a different “canned” experiment each week, while here you carried out various techniques repetitively, but each outcome is different. How does this affect your level of learning or confidence?*

“It is very helpful about learning lab techniques. In other lab classes, we may only have one or two chances to perform one type of experiment, so if you mess up or don’t understand that, well you don’t have chance to go back and try again. But in 187, you can really learn these techniques in depth since you can do them repetitively.”

“Other classes I have taken were very big and vague. They also cover materials that are too broad, so I did not have a chance to learn the techniques thoroughly. This class made me perform techniques by myself and gave me a chance to think deeper about such activities.”

“This lab helped solidify the techniques learned. I easily forget how to perform a lot of the experiments in other labs where it is only presented once; the repetitiveness helped me remember and definitely boosted my confidence as I did everything correctly.”

“I disagree with the statement that our techniques were simply repetitive. Different assignments were assigned each day of the week, then a new set of tasks were given during the fifth week. Each student did not only complete his/her task, but helped others with various tasks. We also worked with different sets of DNA samples each day, and confronted all sorts of possible errors. The variability factor of this research is what enhanced our learning and confidence. The repetition is what fine-tuned our techniques.”

“I enjoyed this lab much more than any other lab I’ve taken. This lab’s experimental procedures although [repetitive], we get constantly different results. And we use critical thinking to figure out what went wrong or what could be done to improve the outcome. This exercised my critical thinking skills, and is a much more worthwhile skill.”

“More practice🡪 more confident. I would rather be an expert in one technique first before moving to the next one. LS 187 lab is the best lab I’ve ever had so far.”

“I enjoy doing the same thing every week. I get to be good at what I do like loading the gel. This is something I don’t get in other classes, each week the labs are so different that I only learn the basic and can never get good at it.”

“I felt that by repeating the experiments every week, it helped me master the techniques needed to carry on the experiments. In other courses, when we performed a different experiment each week, I was never able to master the techniques or really understand them.”

“This lab experience has fine-tuned my abilities, to conduct an experiment more carefully. I am able to meticulously analyze last week’s errors and keep correcting new errors that arise. This repetitive course of action is far more beneficial than “canned experiments.”

“The lab experience in LS187 is more useful than the other courses, because we have the chance to practice the techniques until we are confident in them. And even though we are doing the same things throughout the quarter, we are trained to troubleshoot our research problems, and other courses are not likely to provide this kind of training.”

“I liked the laboratory experience. In other labs, we performed different experiments each week, so it might be a little more interesting, but since we only did them once, I feel like I didn’t completely understand what I was doing. Although our experiments were repetitive, I gained a greater understanding, because I was able to go over the results and see why they may have varied.”

“This gave me a feeling of actually being in a lab because I believe that real research requires repetitive performance of experiments. This also gave me a strong confidence on using the techniques we learned.”

“The lab environment was very comfortable. An ongoing project instead of a different experiment every week kept us on our toes and striving to be more efficient every week. Instead of touching on many different topics and not fully understanding them, we focused on one project and learned about everything about it. We learned more, and repeating many techniques over and over made us more confident and efficient.”

“The repetitiveness of lab allowed us to “Master” the techniques better and to feel more comfortable with our work. In other lab classes, most of the time if you don’t catch a concept the first time around, you luck out – but in this class, there is always tomorrow.”

“The class gives you confidence in your ability to apply book learning to the lab”

“I feel like I can go into a job interview and list some tangible skills, something that is very reassuring for a graduating senior!”

“Poster day was a great opportunity to present our work and “Brag” to our peers.”

“The lab work done in this class is repetitive, but it made me more confident of what I was doing and gave me a purpose.”

“By doing the same things over and over again, I felt an increased level of confidence as well as a higher accuracy when carrying out the task.”

“I feel very confident in the techniques that I carried out each week, and I feel that I thoroughly understand the principles behind PCR, DNA sequencing, and BLAST searches.”

“I definitely enjoyed the lab setting of LS187. It was conducive to learning and self-improvement without being competitive or “cut-throat”. After a couple weeks, I became more comfortable and confident about my abilities.”

“The labs are great. I feel that I’ve learned a lot from all the practices there. Now, I’m so confident about working with DNA sequencing.”

”Initially, I was super nervous doing research, but this class helped me realize I was able to do research.”

“It builds confidence, comprehension and lab coordination skills! Very beneficial and better than traditional lab classes.”

“When I was writing the application for LS187, there was a section for Lab techniques. Although I ended up writing everything from my LS molecular Bio Lab course, I hesitated if I should write those techniques down, because I only performed the techniques one or twice at most and probably not remember how to do them anymore. But now, if someone asks me for previous laboratory experiences and techniques, I can tell that I was involved in the genome sequencing project of *A. degensii* and that I **know** a few techniques.”

“I was doing the techniques on my own and solving the problems on my own.”

“Initially, I was super nervous doing research, but this class helped me realize I was able to do research.”

“Courses like this can bring out the best in a person”

“We learned responsibility and time management in a lab.”

**5. Building teamwork**

*Did you notice any difference between this class and others you have taken in terms of teamwork and collaboration?*

“This class was a lot more fun than the regular lab classes. I was able to work with more people than just one person like in other lab classes.”

“Undergrads running our own research project builds more camaraderie.”

“This class gave me the opportunity to meet great people. I did not have enough opportunity to have deep friendships at school before. We need more opportunities like this so that we can have learning processes through deeper relationships.”

“Basically everyone has to do good work so other students can work after. In other classes, everybody work individually. I think that’s the only course that offers the real teamwork opportunity which I have taken.”

“There’s definitely more teamwork and colleague interaction in this class. We learned to trust one another while at the same time we get to apply what we learn in lecture and lab.”

“In other labs, lab reports tend to be lengthy and require a lab partner to work with. In this lab, your aren’t confined to one lab partner, but everyone in the lab is a potential resource or lab partner in that you can assist them in their work or vice versa.”

“Our lab was like a family helping each other.”

“Very collaborative; my work affects other people’s work and other people’s work affects my work.”

“There was much more closeness among peers here.”

“The class is very much focused on working together which makes things easier and fun.”

“This is the first time I’ve been in a class where everyone is willing to help each other. Before everyone worked on their own because the class is based on a curve.”

“This class definitely relied more heavily on teamwork and collaboration because the data generated depends on the smooth operation of the lab.”

“In other classes I work on labs individually or if it is group work, you only work within your group and are not concerned with other people’s results. In this class, it is so essential that we collaborate and help each other out in order to get accurate results.”

“There is a lot of teamwork. You get to know the professors intimately for a change. I made some new friends. I was able to joke, and act silly/relaxed around my fellow lab mates. I like that you don’t get stuck with one lab partner and you get to know everybody.”

“We all got along really well, and I think it was because we all relied on one another to do a good job.”

“You get real undergraduate teamwork in this class. Unlike other classes, where you see more the intentions of not helping other undergrads in order to get a higher score on a curved class.”

“All the steps involved in this program are interdependent. As the team that we formed, because each of us was responsible for a fundamental step in this chain, we were also like a family. We helped each other, mentored each other, and supported each other when techniques constituted a particularly difficult task to achieve, such as gel loading as far as I am concerned. With time, support, and patience from my fellow classmates… I was finally was able to achieve my goals and master certain lab techniques.”

“LS187 is basically surrounded by a team with great commitment from each individual. I think each of us somehow manages to achieve our goal well to maintain our teamwork.”

“This class requires more teamwork than other traditional lab courses since different people perform different tasks and they are interdependent.”

“It was way better than traditional labs! I have worked in several labs before that; all followed traditional lab methodology. Here, in LS187, you get to freely ask questions to classmates and share ideas to get help from B&C students.”

1. **Making impact on world – Personal significance, Synthesis**

*Are there any other comments you would like to make regarding LS187 or the UCLA Undergraduate Genomics Research Initiative?*

“The feeling that we were doing something never done before and that the results of our work would be utilized by other scientists was a good motivating factor”

“For once I get to be doing something that is actually of importance – instead of other lab classes where you just do the procedures in order to learn it. It’s awesome to know that the data we produce will be used by people in the future. One day, I can say, ‘Hey! I helped sequence a genome!’ ”

“I liked the fact that the results count for not just grades, but actual research data.”

“The fact that our data were being contributed to the worldwide databank motivated me to produce valuable results, since the results were actually being utilized. In contrast to a regular lab class, it usually does not matter if you do not obtain the correct result, since one experiment does not depend on the other. In this course, I not only had to figure out why the experiment did not work, but I also had to redo the experiment since we had a real goal to meet.”

“If I know that whatever I do is going to end up on NCBI, the NCBI website, together with MIT, Harvard results of the Human Genome, I feel responsible for all the results that I produce. They have to be the best I can produce!”

“This made me take my work more seriously - realizing that my work would be deposited in a database + be permanent + not just an experiment in itself.”

“I definitely prefer LS187 over the other lab courses. When I come to class, I actually want to be here. We’re involved in something important, and the techniques we learn can be applied to other courses.”

“Knowing that my work could contribute to the real scientific community made me work harder and try to make less mistakes.”

“I’m always motivated to perform well in lab, but knowing that other scientists might be able to use our findings is pretty cool.”

“To be able to actually see the output of our work contribute to the development of science definitely increased my interest and motivation toward further investigation in the realm of science. In many other labs, experimental results are often not published, so all the hard work is often unrecognized. However, knowing that our work here at LS187 contributes directly to NCBI is a very gratifying experience.”

“Definitely, I mean to some extent, it did encourage me to put more work in it since I know it’s a big research, one of a kind for undergrads and the scientific community might use our publicly online displayed data. It also motivated LS3 students when I told them so.”

“I am doing research for the WORLD!! O.M.G!!

“I feel that in other lab courses, the experiments have preordained outcomes, but they have the specific purpose of teaching the basics of the principles upon which everything else relies upon. However, in comparison to these courses, LS 187 shows us the possible exceptions, either due to human error or equipment error. The increased chances of these occurrences help emphasize the importance of proper lab techniques, stressing accuracy and precision.”

“Part of what made this class so great was the background of the hands-on learning: either a class will focus only on hands on (i.e. chemistry labs) or only on learning – but the delicate balance of the two makes the class all the more unique and worthwhile.”

“I have learned a lot of techniques and skills that would remain with me for a long time rather that just following a protocol in an experiment in a Life Science class.”

“In other classes, you only do things once and usually we just follow directions step-by-step. In this class, I liked that we didn’t just follow directions because we had to modify it sometimes based on trying to make improvements.”

“I feel I’ve gained more experience from this lab than from others, because I really understand the theory behind our results. In other classes, I sometimes feel like I just follow directions, and hope my results turn out correctly.”

“This was a totally unique class. I like that you repeated protocol procedures, which helped me develop a greater competence for laboratory techniques. I was also able to witness the large scale chain of events, i.e. how one day’s work connected to another’s, unlike disjoint LS labs. I became more confident and learned more (and wanted to learn more) in 187A than any other lab class.”

“I feel very fortunate to have been able to participate and to contribute the UGRI. I hope labs (Classes) like this increase so that the undergraduates can find science exciting and stay. That will allow students to see research as a profession, not just a means to get to medical school.”

“I liked 187 much more than my other lab courses, because were actually doing something important, not just performing experiments with a known outcome. Also, I really learned about the procedures used, which most likely I won’t forget.”

“This course taught me the value of teamwork, discipline, time management and great knowledge.”

“When a student knows the results of the lab beforehand, no matter what he/she does, the result is predictable. Not in all people, but this “known end” can bore a lot of people and besides, the “perfect result” is on the T.A.’s desk anyway, so what’s the point?”

“I like the fact that undergraduates are running their own research project, because it gave me the opportunity to feel in control of what I was doing and to fully understand the whole process.”

“I think the biggest strength of this lab was getting exposure to an experience I have never had before. I was able to learn what it was to be like a researcher. It was very fun. I do not think there really was a weakness. I believe as the class goes on we became more skilled. Maybe giving us more responsibility would be nice.”

“Compared to my other lab courses, LS187 was the most significant in my scientific career. I not only gained very valuable experience, but I was held accountable for my work which really forced me to pay attention and to take pride in everything I do. Additionally, LS187 is the only lab course where I actually knew what was going on.”

“LS187 makes UCLA a greater ‘research’ university for undergraduates.”

“I think this has been one of the most valuable experiences I have participated in at the university.”

“It would be great for undergraduates if this kind of class was expanded to allow even more students to participate.”

“I am definitely grateful to be a part of such a singularly unique class and truly feel that the depth of understanding required and the theory discussed are a meaningful way to create well-equipped students for further research.”

“I am fortunate enough to be part of this project and I believe that undergraduates are just as smart as graduate students. LS187 is a very valuable resource for students like us who are undergrads and need good experience.”

“I wish all the luck to the Initiative and all the people involved in it. You are pioneers in trusting undergraduates with important research that would yield important results.”

“I would totally recommend it. 1^st^🡪knowledge and application of knowledge in real world applications. 2^nd^🡪 make great friends.”

“I love it, and think there should be more research-based classes like it.”

“It was a truly priceless learning experience. I had a great time and was reminded for the first time in a long time, that biology could be fun and more than just constant remedial memorization. It was great to be able to truly physically apply what I learned and to also be able to constantly follow up with results and mistakes.”

“We should have more classes like LS87, because I really learned something useful from the class”

“More than a cog in a lab, I am a thinking participant in the research process.”

“It is quite a challenge, but giving this opportunity to undergraduates is an invaluable opportunity. It teaches personal accountability and is able to truly demonstrate what a ‘real-life’ lab would demand.”

I enjoyed this course very much. I was able to master the techniques taught in this course because I had to teach them to other students. That is what was unique about this class. I was able to learn, teach, and produce novel information. I would definitely take this class again and recommend this course to hardworking students especially those interested in research. I am very lucky and happy to be a part of this course and its project. This as truly a remarkable experience; a class unlike others.
